# Introducing Holographic Surgical Navigation in Pediatric Wilms’ Tumor Patients: A Feasibility Study During Total Nephrectomy

**DOI:** 10.3390/bioengineering12080896

**Published:** 2025-08-21

**Authors:** Nick T. de Groot, Jasper M. van der Zee, Guus M. J. Bökkerink, Annemieke S. Littooij, Caroline C. C. Hulsker, Cecilia E. J. Terwisscha van Scheltinga, Cornelis P. van de Ven, Ruud C. Wortel, Aart J. Klijn, Marc H. W. A. Wijnen, Matthijs Fitski, Alida F. W. van der Steeg

**Affiliations:** 1 Department of Pediatric Oncology Surgery, Princess Maxima Center for Pediatric Oncology, 3584 CS Utrecht, The Netherlands; 2 Department of Radiology and Nuclear Medicine, University Medical Center Utrecht/Wilhelmina Children’s Hospital, 3584 EA Utrecht, The Netherlands; 3 Department of Pediatric Urology, University Medical Center Utrecht/Wilhelmina Children‘s Hospital, 3584 EA Utrecht, The Netherlands

**Keywords:** augmented reality, HoloLens 2, landmark-based registration, nephron-sparing surgery, total nephrectomy, Wilms’ tumor

## Abstract

Wilms’ tumor is a common pediatric renal malignancy. In selected cases, nephron-sparing surgery (NSS) may be employed as part of the surgical approach. To prevent positive margins, optimal understanding of the tumor–kidney edge is essential. Augmented reality (AR) enables intraoperative visualization of patient-specific three-dimensional (3D) holograms. In this study, we aim to validate the clinical feasibility of a holographic landmark-based registration system in pediatric patients planned for a total nephrectomy (TN), to ensure that the holographic visualization will not influence surgical decision making. In a single-center prospective study, ten pediatric patients undergoing TN were included. Patient-specific 3D holograms were created from preoperative MRI, and intraoperatively landmark-based registration was performed using the HoloLens 2. Clinical feasibility was conducted using accuracy measurements, the System Usability Scale (SUS), and a self-developed questionnaire. Three out of ten patients had a successful registration with a median measured accuracy of 7.0 mm (Interquartile Range (IQR) 6–13.5) and a median SUS score of 75 (IQR 65–77.5). Surgeons reported improved depth perception and anatomical understanding. However, in seven out of ten patients, registration failed due to multiple reasons. The most important factors were large tumor volumes obstructing landmark placement and insufficient spatial distributions of the landmarks, causing rotational misalignment. Although AR showed potential in improving the depth perception and relation in anatomical structures, the landmark-based registration with the HoloLens 2 was currently deemed insufficient for clinical implementation in pediatric abdominal surgery.

## 1. Introduction

The most common childhood kidney tumor is Wilms’ tumor (WT), with an annual incidence of 0.7 in 100,000 children younger than 15 years. It carries a good prognosis, with overall survival rates of over 90% [1,2]. Surgical management involves either a total nephrectomy (TN), entailing complete removal of the affected kidney and tumor, or nephron-sparing surgery (NSS), in which the tumor is excised while preserving uninvolved renal parenchyma. The goal of NSS is to preserve functional renal tissue whilst achieving a complete tumor resection [3]. This surgical treatment is considered for bilaterally affected patients and patients with a genetical predisposition for WT. Unfortunately, between 15.7% and 31% of the tumors are incompletely removed, a so-called positive surgical margin [4,5]. For these patients, the treatment protocol advocates additional chemotherapy and radiotherapy.

To reduce positive surgical margins, a detailed intraoperative understanding of the anatomical relationships of each specific patient is vital. Novel surgical visualization techniques can help to gain an improved understanding of the three-dimensional (3D) anatomical relationships of the crucial structures of the kidney such as vasculature, urinary collecting system, and tumor localization and infiltration [6]. Such anatomical relationships can be shown in 3D both pre- and intraoperatively with a HoloLens 2 [7]. The HoloLens 2, an augmented reality (AR) head-mounted display, visualizes holograms of the patient’s anatomy for preoperative planning. Moreover, AR can also be used to visualize holograms during surgery onto the patient. By correlating preoperatively defined anatomical landmarks with intraoperative target landmarks, we can register the hologram on the true location in the surgical field. The method behind this technique has been described in an earlier study visualizing Ewing sarcoma on the body surface [8,9]. The preliminary results of a technical validation in a phantom study for kidney tumor visualization resulted in a mean registration accuracy of the holograms of 3.16 ± 1.82 mm. This technical validation confirmed that the algorithm worked as technically expected, within the clinically determined error margin (<5 mm).

Nevertheless, the environment during open kidney tumor surgery inside the operating room is vastly different from the phantom environment during technical validation. This includes additional discomfort while wearing the HoloLens, additional difficulty to pinpoint the required points and, most notably, a more deformable kidney. The effect of these real-world differences of the procedure needs to be studied, as they can influence the accuracy of holographic overlay. Moreover, we need to ensure that the preparation of the technique is feasible and consistent within the clinical workflow. These differences need to be studied before implementation of the technique during NSS.

The objective of this feasibility study was to evaluate the landmark-based registration with the HoloLens for inside the operating room on patients undergoing a TN.

## 2. Materials and Methods

In this clinical mono-center prospective feasibility study, we included all patients (n = 10) aged between 6 months and 18 years with a renal tumor undergoing a TN between 1 December 2023 and 1 May 2025 in the Princess Máxima Center for Pediatric Oncology. We included eight male and two female patients, with a median age of 48 months (Interquartile Range (IQR) 29.5–55.5 months). All patients received preoperative chemotherapy according to the UMBRELLA protocol [2]. Patients or their parents were verbally informed before inclusion. The study did not fall under the scope of the Medical Research Involving Human Subjects Act, as assessed by the Medical Ethical Review Committee (research proposal 23-145/DB).

To continue clinically towards NSS, we have set three specific objectives for this feasibility study:

1. The error of the accuracy of the registration of the hologram reaches a median accuracy of less than 5 mm;
2. The System Usability Scale (SUS, range 0–100) should have a median of 60 or higher to confirm usability and comfort for the surgeons;
3. The technique should be consistent and accurate. Therefore, in this study, we required five successful registrations within 10 min in five consecutive patients.

Furthermore, information was gathered on clinical patient characteristics, including diagnosis, oncologic treatment, surgical indication, and radiographic parameters. The preoperative MRI of the patient was used to create the patient-specific hologram.

### 2.1. Study Procedure

#### 2.1.1. Preoperative Planning

Preoperatively, the 3D model of the patient was segmented based on standard preoperative MR imaging acquired with a 1.5 Tesla system (Ingenia, Philips Medical Systems, Best, The Netherlands), using the Mimics Innovation Suite 26.0 (Materialise, Leuven, Belgium), following the workflow described by Fitski et al. [10]. The radiologic tumor’s size was calculated by multiplying the tumor height, length, and width and then multiplying the result by 0.523. The 3D model included the kidney, tumor, renal arteries, and veins. Subsequently, the holographic matching software was prepared in Unity 2019.4.22.f1 (Unity Software Inc., San Franciso, CA, USA) by defining different anatomical landmarks. The preoperative anatomical landmarks included the cranial side of the kidney, the lateral side, the caudal side, the anterior side, the hilum, and the bifurcation of the renal arteries from the aorta or of the renal veins from the vena cava.

#### 2.1.2. Intraoperative Protocol

**Figure 1:**
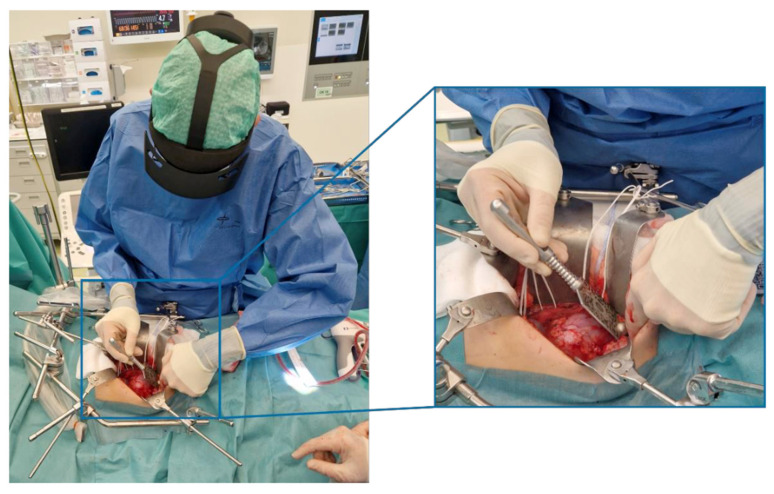
Intraoperative registration process by pin-pointing anatomical landmarks using a sterilized QR code.

The TN started with the regular surgical protocol, freeing the kidney with the tumor from the surrounding tissue. Then, one of the five participating surgeons, who all received training using a phantom, selected five anatomical landmarks in the holographic application for landmark-based registration on the HoloLens 2 (Microsoft Corporation, Redmond, USA). A sterilized pointer with a quick-response (QR) code enabled the HoloLens to track the spatial location of the pointer. Using the pointer, the surgeon identified each target landmark and placed the corresponding virtual points via a voice command (Figure 1). Once all five anatomical landmarks were defined, the system used a Procrustes algorithm to align the preoperative hologram with the patient’s anatomy. A separate reference QR code was used as a stable visual anchor to maintain the position of the hologram within the surgical field. This reference QR code was placed in the sterile operating field, but neither in the surgical wound nor the QR code was fixated to the kidney.

A registration was considered successful when the hologram overlayed with the correct orientation. In the event of a registration failure, we documented the specific cause of the registration error. The surgeon observed the resulting holographic visualization carefully. Secondly, the surgeon measured the difference in the position of the edge of the kidney with the edge of the holographic overlay using a sterile measuring ruler. During the procedure, we measured the complete time of the holographic visualization. After measurements, the surgeon continued with the conventional surgical procedure and resected the kidney with tumor from the patient.

#### 2.1.3. Postoperative Analysis

Postoperatively, the surgeon was asked to complete two questionnaires, the SUS and a self-developed questionnaire to qualitatively assess the feasibility, comfort level of the surgeon, the user-friendliness, and the observed clinical accuracy of the technique [11]. A SUS of <50 is considered not acceptable, 50–70 is marginal, and >70 is acceptable [12]. The self-developed questionnaire can be found in Table 1 in the Results section. The questionnaires were completed after a successful intraoperative registration of the hologram.

### 2.2. Data Analysis

All data were anonymized, and descriptive statistics were determined in IBM SPSS Statistics 29.0.2.0 (IBM, Armonk, NY, USA). This study was descriptive in nature; therefore, no inferential statistical analysis was conducted.

## 3. Results

### 3.1. Patient Characteristics

**Figure 2:**
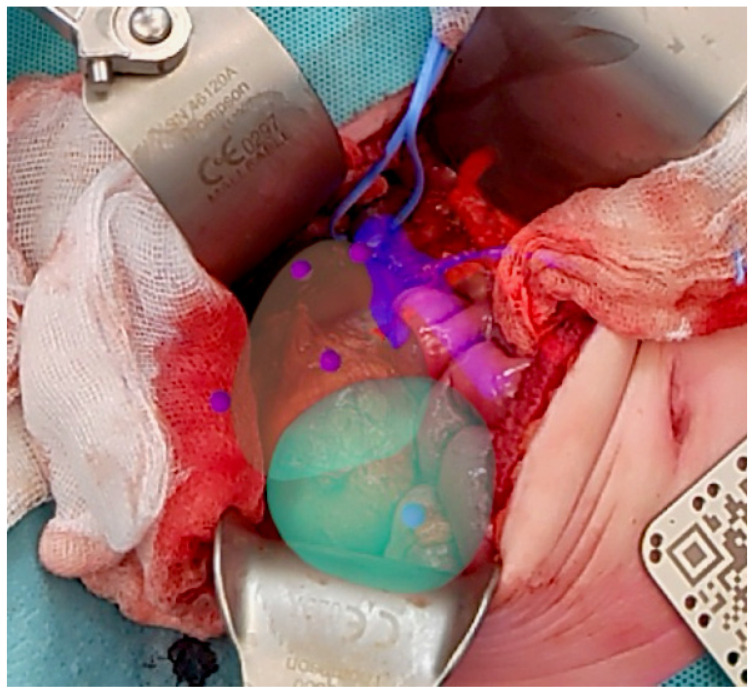
Intraoperative photo of a successful registration with the HoloLens 2 during a TN of a WT. The kidney overlay is transparent, tumor is visualized in turquoise, and artery and veins in red and blue, respectively. Notice how the upper part of the vena renalis visualization is entangled by vessel loop. The target landmarks used by the algorithm are shown in purple. The sterile QR code is used as a visual anchor to stabilize the hologram.

In total, we included ten patients with a median tumor volume at time of surgery, based on radiologic measurements, of 115.5 mL (IQR 45.5–220.25). In 3/10 cases, a successful intraoperative registration of the hologram was achieved. Figure 2 shows an intraoperative photo displaying the holographic overlay after a successful registration.

### 3.2. Quantative Results

In case of a successful registration, the registrations had a median duration of 11 min 30 s (IQR 8.25–13.25). The overlay demonstrated a median measured accuracy of 7.0 mm (IQR 5–13.5). Surgeons subjectively rated the accuracy with a median score of six out of ten (IQR 5–6.5). The SUS had a median score of 75 (IQR 65–77.5).

### 3.3. Qualitative Results

All results of the self-developed questionnaire can be found in Table 1. There were discrepancies among the surgeons regarding whether identifying target landmarks on the kidney was a straightforward task. However, they consistently agreed that, if the holographic overlay was successfully aligned, the holographic overlay increased the depth perception and the understanding of anatomical relationships in the patient. In particular, the holographic visualization of the tumor and surrounding vessels was considered potentially valuable for NSS. Nevertheless, opinions differed on whether the benefits of the holographic overlay justified the additional time required for its implementation.

**Table 1:** Results of the self-developed questionnaire concerning the usability of the holographic registration technique in cases of successful registration. All questions were scored from 0 (totally disagree) to 10 (totally agree).

#	Question	Case 1 (Surgeon 4)	Case 2 (Surgeon 2)	Case 10 (Surgeon 1)	Median Score (IQR)
1	Determining the position of the target landmarks on the kidney of the patient was straightforward.	7	2	8	7 (4.5–7.5)
2	The user interface of the HoloLens software was straightforward to use.	8	8	8	8 (8–8)
3	The hologram remained stable on the kidney after positioning.	10	4	10	10 (7–10)
4	The holographic overlay improves my sense of depth.	7	8	8	8 (7.5–8)
5	The holographic overlay improves my understanding of the anatomical relationships of the patient.	8	8	10	8 (8–9)
6	The holographic overlay visualized the position of the intraparenchymal vessels reliably.	7	Not defined, as vessels were not present in 3D model	8	7.5 (7.25–7.5)
7	The holographic overlay of the tumor and vessels is useful for NSS.	8	Not defined, as vessels were not present in 3D model	8	9 (8.5–9.5)
8	The holographic overlay is worth the additional time for NSS.	8	5	10	8 (6.5–9)

### 3.4. Observed Causes for Non-Successful Registration

Intraoperative registration was unsuccessful in seven out of ten procedures. While there were different reasons, two reasons were most common. We observed that in three patients, the intraoperative tumor volume was so large, it was regarded as unsafe or impossible to position the target landmarks. This was the case when the tumor extended across the midline or towards the diaphragm. Therefore, several landmarks were considered inaccessible, and the point registration procedure was not performed. In two cases, we the surgeons could not position the most caudal and cranial target landmarks without moving the kidney. Additionally, due to the difficult depth positioning of the target landmarks, all landmarks were approximately in the same plane. This resulted in a wrong positional matching of the Procrustes algorithm, leading to a rotational error. This could not be corrected for during re-registration.

## 4. Discussion

This was the first study to use the Procrustes registration algorithm to rigidly position holograms in the operative field during a TN in children. It aimed to improve the detailed understanding of the anatomical relationships of the patients to reduce positive surgical margins. Unfortunately, the feasibility was unsuccessful, as we did not reach the objectives of this study because we could not achieve adequate registration in five consecutive patients.

Previous studies have shown more favorable results in employing AR for surgical navigation [7,13]. However, landmark-based registration studies show similar accuracy measurements. Konovalov et al. used a cranial landmark-based holographic alignment method, which showed that an external ventricular drain placement had, in multiple cases, deviation 0.5 mm to 2.3 mm in comparison with the planned trajectory [14]. Van der Woude et al., who used the same holographic navigation system but for Ewing Sarcoma, showed an accuracy of mean Target Registration Error (TRE), which is the distance between corresponding target points after registration, of 6.2 mm in phantom experiments [9]. Although our results seem less favorable, direct comparison is challenging. The applications of both Konovalov and Van der Woude primarily involve rigid anatomy and pre-incision applications, which present less difficult challenges compared to intraoperative abdominal conditions where factors such as organ movement, soft tissue deformation, and visibility may hamper accurate holographic visualization.

Recent advancements in laparoscopic NSS in adults have introduced solutions to overcome above-mentioned challenges in abdominal holographic visualization [15]. Piana et al. introduced a machine learning approach that aligned preoperative 3D models to real-time endoscopic images, even accounting for kidney rotation and deformation [16]. Noticeably, laparoscopic imaging provides more geometric and visual information than current head-mounted AR devices, which enables the development of more accurate and automated tracking algorithms to superimpose 3D models [14,17,18].

Clinical studies are demonstrating the surgical effectiveness of 3D models during surgery. For instance, Minchiels et al. found that 3D virtual models without AR overlay in robot-assisted partial nephrectomy led to a lower rate of major postoperative complications, improved preservation of renal function, and reduced positive margins, even in cases with more complex tumors [19]. Secondly, Porpiglia et al. found that manually superimposing 3D models on endoscopic images may improve the quality of the resection phase and reduce postoperative complications, with better functional recovery [20].

It is apparent that our holographic navigation technique did not work in the operating room as expected based on the technical preclinical validation. While we expected differences between the technical validation and clinical application, the observed differences were much larger than anticipated. Several technical and clinical reasons were observed during this study. Firstly, in the operating room, differences in reflections caused by the moisture on tissue and surgical tools meant that the HoloLens 2 tracking accuracy was reduced. The environmental mapping of the HoloLens 2 was potentially less stable, requiring more computational power, and the QR tracking of the surgical tool became less accurate. Secondly, we developed the technique for NSS. However, we included patients undergoing TN to prevent the holographic visualization from affecting the surgical outcome. Patients in this group typically have a larger tumor volume than tumors eligible for NSS, and the free abdominal space was smaller. Fitski et al. reported a median tumor volume of 2.5 mL (IQR 0.75–19.5) in a cohort of patients undergoing NSS, which is much smaller than the median volume of 115.5 mL (IQR 45.5–220.25) in this study [6]. This made it harder to reach the anatomical landmarks on the outside of the kidney, most of the time moving them more medially. Subsequently, the difference in height between the landmarks was reduced, limiting the three-dimensional features of the landmarks. This resulted in inaccurate rotations of the hologram. In specific cases, we could not reach all anatomical landmarks without moving the kidney during registration, and the registration was not performed. Secondly, when the intraabdominal pressure is relieved during surgery, larger tumors may bulge out, causing a difference in tumor volume and rotation of the kidney. Again, we suspect this limited the positioning accuracy of the landmarks. We conclude that landmark registration algorithms are currently not sufficient for intraoperative abdominal visualization techniques. 

It is difficult to measure the intraoperative accuracy of the hologram [7,13]. We used a measurement of the surgeon by measuring the difference between the tumor–kidney border in the hologram and the actual tumor–kidney border. Our measurement did not allow for a measurement of the TRE. The static location measurement does not take the rotational and scaling error into account. The border may be measured accurately, while the rest of the visualization is distorted. In future work, we should include TRE measurements, which would provide us with more insights into the translational and rotational inaccuracies. Secondly, we should evaluate the accuracy of the holographic overlay by comparing it with intraoperative ultrasound. To do this, we can mark the resection border of the holographic overlay and compare the distance with the marking of the intraoperative ultrasound.

This clinical feasibility study was meant to validate our intraoperative registration technique. Unfortunately, the technique is considered inaccurate, and we will not proceed with the current holographic system for further applications in either TN or NSS. However, we have identified several technical requirements that will be necessary for further clinical implementation, such as real-time tracking, compatibility with smaller surgical tools, and a stable system with minimal drift to eliminate the need for additional anchoring.

To address these requirements, several technological advancements should be considered. Firstly, the size of tracked surgical tools needs to be reduced. In this study, we used a five-by-five cm QR code pointer, but infrared or electromagnetic markers for pointer tracking may be more suitable. These markers can be significantly smaller than the currently used pointer [21]. Secondly, surface mapping of the kidney surface combined with an iterative closest point registration algorithm might be more suited for this problem [22]. This technique uses many more landmarks for registration, limiting the effects of a low intraoperative landmark registration error. Thirdly, as support for the HoloLens 2 has recently been discontinued by Microsoft, we need to look past this device also.

Additional to these aforementioned techniques, there will be two major technical advancements relevant for nephron-sparing surgery. With the emergence of advanced AR devices, it becomes possible to obtain more data with advanced sensors such as depth and high-resolution cameras. This can be used to overcome challenges caused by deformability and movement. Real-time deformable object tracking algorithms could be studied, such as in laparoscopic surgery, exploiting deep learning algorithms but for open NSS [23,24]. Additionally, intraoperative tracked ultrasound can derive features required for accurate registration. Tracked ultrasound may be combined with AR visualization to exploit the benefits of both techniques [25]. Both solutions may be considered harmless and should be considered for further development in this field.

## 5. Conclusions

In conclusion, the clinical implementation of intraoperative hologram projection using the HoloLens 2 for open TN, based on landmark registration, proved unsatisfactory. Nevertheless, in three of the ten cases with successful registration, the system enhanced anatomical understanding and depth perception, highlighting the potential of AR in surgical navigation. Limitations in intraoperative landmark localization and tracking instability adversely affected registration accuracy and overall clinical usability. Future developments must focus on automatic and robust registration methods, potentially using more advanced AR devices combined with more and different sensor data.

## Data Availability

The raw data supporting the conclusions of this article will be made available by the authors on request.

## Abbreviations

The following abbreviations are used in this manuscript:

3D	Three-dimensional
AR	Augmented reality
IQR	Interquartile Range
MRI	Magnetic Resonance Imaging
NSS	Nephron-sparing surgery
QR	Quick Response
SUS	System Usability Scale
TN	Total nephrectomy
TRE	Target Registration Error
WT	Wilms’ tumor
